# Relevant HRH leadership during public health emergencies

**DOI:** 10.1186/s12960-022-00723-2

**Published:** 2022-03-24

**Authors:** Paulo Ferrinho, Uta Lehman, Eszter Kovacs, Mario Dal Poz

**Affiliations:** 1 Research Centre on Global Health and Tropical Medicine, Instituto de Higiene e Medicina Tropical, Universidade Nova de Lisboa, Rua da Junqueira 100, 1349-008 Lisbon, Portugal; 2 Present Address: School for Public Health, University of Western Cape, Cape Town, South Africa; 3 Present Address: Semmelweis University-Health Services Management Training Centre, Budapest, Hungary; 4 Present Address: Universidade do Estado do Rio de Janeiro-Instituto de Medicina Social, Rio de Janeiro, Brazil

**Keywords:** Leadership, Health labor market dynamics, Human resources for health, Human resources impact assessment, COVID-19, Pandemic, Public health emergencies

## Abstract

**Background:**

Inadequate leadership capacity compounds the world's workforce lack of preparedness for outbreaks of all sizes, as illustrated by the COVID-19 pandemic.

Traditional human resources for health (HRH) leadership has focused on determining the health workforce requirements, often failing to fully consider the unpredictability associated with issues such as public health emergencies (PHE).

**Main arguments:**

The current COVID-19 pandemic demonstrates that policy-making and relevant leadership have to be effective under conditions of ethical uncertainty and with inconclusive evidence. The forces at work in health labor markets (HLM) entail leadership that bridges across sectors and all levels of the health systems. Developing and applying leadership competencies must then be understood from a systemic as well as an individual perspective. To address the challenges described and to achieve universal health coverage (UHC) by 2030, countries need to develop effective HRH leaderships relevant to the complexity of HLM in the most diverse contexts, including acute surge events during PHE. In complex and rapidly changing contexts, such as PHE, leadership needs to be attentive, nimble, adaptive, action oriented, transformative, accountable and provided throughout the system, i.e., authentic, distributed and participatory. This type of leadership is particularly important, as it can contribute to complex organizational changes as required in surge events associated with PHE, even in in the absence of formal management plans, roles, and structures. To deal with the uncertainty it needs agile tools that may allow prompt human resources impact assessments.

**Conclusions:**

The complexity of PHE requires transformative, authentic, distributed and participatory leadership of HRH. The unpredictable aspects of the dynamics of the HLM during PHE require the need to rethink, adapt and operationalize appropriate tools, such as HRH impact assessment tools, to redirect workforce operations rapidly and with precision.

## Background

The COVID-19 pandemic has challenged every dimension and every level of health systems around the world, bringing their weaknesses and failings into sharp relief, and focusing attention on the need to review and adapt how health systems function and are governed. This is particularly true for human resources for health (HRH), who have been at the center of the pandemic response internationally, highlighting the relevance of WHO’s “Global Strategy on Human Resources for Health: Workforce 2030” policy options and recommendations for transformative actions to tackle emerging HRH challenges towards attaining universal health coverage (UHC).

As COVID-19 incidence and prevalence rose in different countries at different times, several factors, important especially in low- and middle-income countries, but not uncommon in the high-income countries, gained prominence: inequitable distribution of the HRH, poor quality of training, lack of supervision, imbalances in skill-mix and task shifting, lack of basic working conditions to support adequate productivity and performance, low retention and high turnover, lack of preparedness to ensure the safety and well-being of frontline HRH, hence, accentuating long-standing inadequacies in responding to global and national health labor market (HLM) dynamics [1].

These dynamics are influenced by seemingly predictable factors (e.g., the health needs of the population, the demand for health services and the supply, demand and governance of health workforce). Traditional HRH leadership has focused on determining the health workforce requirements to address these factors, often failing to fully consider the unpredictable dynamics associated with issues such as public health emergencies (PHE) [2].

Traditional HRH leaderships have failed to balance health-policy developments that respond to “the pressure of urgent requirements that are not always amenable to a long-term approach”, with “investments and interventions regarding human resources for health” that may show results only in the medium and long term, setting the foundations of preparedness and resilience [3]. Achieving this balance requires the acknowledgment that HLM dynamics require fast learning, systems agility and inter-sectoral collaboration.The forces at work in HLM demand leadership that bridges across sectors and all levels of the health systems [3, 4].

Developing and applying leadership competencies must then be understood from a systemic as well as an individual perspective. To address the challenges described and to achieve UHC by 2030, countries need to develop effective HRH leaderships relevant to the complexity of HLM in the most diverse contexts, including acute surge events during PHE [2, 5, 6].

Where that does not happen, leadership shortcomings become significant factors of the failure of many countries to respond adequately to the evolving pandemic.

### Key workforce issues during public health emergencies

PHE place extra demands on HRH. HRH leaders are confronted with complex HRH issues that need prompt responses to, namely: increased workload (working longer hours or more shifts), many times under substandard conditions of safety; increased absenteeism for medical and non-medical reasons; inadequate recognition and compensation for work performed; working off-site; changes from usual role requiring additional skills; requirements to work with other sectors and other agencies in a coordinated way (e.g., the army, residential homes for the elderly, international agencies); negative impact on health and well-being of HRH and their loved ones (fear, mortality, morbidity, discrimination, violence, and burnout); ethical dilemmas (associated with triage arrangements, neglect of treatment of conditions that would normally be covered, conflicting loyalties to job versus family and community obligations); working often with insufficient resources, under conditions of disruptions within the public health system and delays in supplies (e.g., personal protective equipment, ventilators, vaccines, and other resources required to provide effective care to patients); dealing with lability of trust. All this, frequently, in a context of lack of data on HRH and under a significant level of scientific uncertainty that place epistemological challenges to HRH in general and physicians in particular [2, 5–8].

Even in non-pandemic situations, reallocation of resources away from specific clinical services has been found to entail negative responses among healthcare staff due to perceived threats to their professional identity. This is reinforced during resource reallocation in acute surge events, contributing to feelings of disempowerment. By the end of the surge public health event, rebounding to their specific clinical duties is not necessarily associated with a return to business as usual because, inter alia, of the need to address issues of preparedness and resilience in preparation for future events [5–7].

### The importance of distributed and participatory HRH leadership

Most leadership theories stemmed from a business context and were adapted to the health sector. They evolved from early theories focused on the traits or innate qualities of the leaders to situational theories in the 1960s expanding the theoretical focus to include the context in which leadership takes place [9].

Authentic leadership theory emphasizes the values system of leaders. Builds on transformational theory, includes elements of charismatic leadership theory and adds a values orientation. A number of studies demonstrated that the authentic leadership theory is particularly applicable to healthcare settings [10–14].

Collective/Shared/Distributed Leadership theories build on the situational approaches to argue that “no one individual is the ideal leader in all situations or circumstances and that leadership is diffuse throughout the organization. Includes dispersed, collaborative, collective, devolved, relational, democratic, concurrent, and cooperative approaches”. These have “been positively correlated with increased team effectiveness and organizational performance” with “demonstrated applicability in healthcare settings and has been adopted by the National Health Service in the United Kingdom” [9, 15, 16].

During PHE the HRH leadership appropriate at different levels of the health system, both within countries and globally, may differ and must address moral, technical and professional skills across all aspects of HRH development and management, to ensure effective policy dialogues, strengthening capabilities to facilitate, direct, motivate and oversee strategic change in and across different dimensions of HRH development and performance as well as operational efficiency. The current COVID-19 pandemic also reiterated that policy-making and relevant leadership have to be effective under conditions of ethical uncertainty and with inconclusive evidence [8]. These understandings require translation into interventions to build leadership capacities for different actors with HRH responsibilities.

Definitions of leadership developed by WHO and the Alliance for Health Policy and Systems Research, as well as understandings of strategic and distributed leadership in organizations, emphasize that, in complex and rapidly changing contexts, such as PHE, leadership needs to be attentive, nimble, adaptive, action oriented, accountable, transformative and provided throughout the system (distributed and participatory leadership) [3, 4, 17]. This has been demonstrated during the emergency phase of the 2014–2015 Ebola epidemic in Liberia. [11].

Distributed leadership is particularly important, as it can enable complex organizational changes as required in surge events associated with PHE, even in in “the absence of formal management plans, roles, and structures” [18]. It requires “transdisciplinary learning and real-world awareness”, that takes into consideration ethical concerns, gender issues, and other sectors’ demands (e.g., the economic and financial sectors, education, sports, cultural and leisure activities, religious worshiping and foreign affairs) on the attention of political decision-making [4].

PHE require a workforce leadership not only distributed, but also participatory, system-wide, among policy-makers and planners, public and private sector employers, professional associations, education and training institutions, labor unions, bilateral and multilateral development partners, international organizations, civil society and health care workers themselves. Within each group of actors, individual leadership (formal and informal) does matter and can be of high value in fostering open, consultative processes of democratic decision-making to bring out the collective strength of participatory leadership, which should also contribute to “mechanisms for mounting disruptive challenges to the status quo as well as system stabilizers, further enabling the health system to withstand internal or external turbulence, creating a dialectical process to guide the debate to clarity of ideas and consensus on actions” [4]. In these contexts, authentic leadership is crucial. This type of leadership is needed to help the HRH to build resilience, trust and responsiveness that can reduce the negative impacts of crises [7, 19].

With the pandemic, it became obvious that such sagacious leadership is not bestowed in one individual or a single institution. It is the shared strength of wide-ranging social systems, including the health care system, that must generate the quality and consistency required to raise the performance of the relevant social systems high above the complexity erroneously interpreted as confusion [4].

Relevant HRH leadership during PHE implies acknowledging that change can be understood but it is not always predictable, occurring under a set of circumstances that many times cannot be anticipated. Under these circumstances, the ability to process and analyze a complex situation in an orderly manner is an essential element of effective leadership. Hence, leaders need skills, mindsets, capabilities and tools that go beyond the usual dimensions of HRH policy and management. These tools, namely human resources impact assessment (HRIA), have been suggested in past publications [20]. Based on the HLM framework and good practice principles from other fields, a draft HRIA tool has been proposed but still needs finalization and validation before it is adopted. Once adopted, it may require further conceptual evolution to accommodate multiple uncertainties [17] and methodological developments to be fully operational and readily applicable to PHE [21].

## Conclusions

This commentary identifies leadership as a complex, multi-contextual process. No single theory provides a satisfactory leadership framework to respond to PHE, identifying the need for rigorous research on the topic. Nevertheless, on the basis of the range of theories on leadership and the limited available empirical evidence, we argue that the complexity of PHE requires transformative, authentic, distributed and participatory leadership of HRH.

Major, and often neglected bottlenecks that add to the complexity during PHE, are issues usually related to unpredictable aspects of the dynamics of the HLM, its impact on health systems’ organization and unmet information needs for evidence-informed decision-making. Hence the need to rethink, adapt and operationalize appropriate tools, such as HRH impact assessment tools, to redirect workforce operations rapidly and with precision.

## Data Availability

Not applicable.

## Abbreviations

HLM: Health labor market
HRH: Human resources for health
PHE: Public health emergencies
UHC: Universal health coverage
WHO: World Health Organization
